# Phytochemical characterization and anti-arthritic potential of green-synthesized CuO nanoparticles derived from the *Bistorta amplexicaulis* root extract

**DOI:** 10.3389/fphar.2024.1474592

**Published:** 2024-12-17

**Authors:** Mahrukh Babar, Bilal Aslam, Muhammad Naeem Faisal, Abdul Malik, Suhail Akhtar, Sabiha Fatima, Wafa Majeed, Asher Umer, Muhammad Akmal Farooq

**Affiliations:** ^1^ Institute of Physiology and Pharmacology, University of Agriculture, Faisalabad, Pakistan; ^2^ Department of Pharmaceutics, College of Pharmacy, King Saud University, Riyadh, Saudi Arabia; ^3^ Department of Biochemistry, A.T. Still University of Health Sciences, Kirksville, MO, United States; ^4^ Department of Clinical Laboratory Science, College of Applied Medical Sciences, King Saud University, Riyadh, Saudi Arabia; ^5^ Department of Pharmacy, University of Agriculture, Faisalabad, Pakistan

**Keywords:** rheumatoid arthritis, green synthesis, copper oxide nanoparticles, *Bistorta amplexicaulis*, anti-inflammatory activity, phytochemical analysis, autoimmune diseases

## Abstract

**Introduction:**

Rheumatoid arthritis is an autoimmune disease that mainly causes joint damage. The patient experiences loss of appetite, pain, fever, and fatigue. The present study was designed to phytochemically characterize and evaluate the anti-arthritic activity of green-synthesized copper oxide (CuO) nanoparticles (NPs) using the hydroalcoholic extract of *Bistorta amplexicaulis* roots in an adjuvant-induced arthritic rat model.

**Material and Methods:**

For this purpose, crude powdered plant material was used for proximate analysis, and the plant extract was assessed for qualitative phytochemical analysis, mineral contents, and flavonoid and phenolic contents, as well as quantitative phytochemical analysis through reversed-phase high-performance liquid chromatography (RP-HPLC) and Fourier-transform infrared (FTIR) spectroscopy. The *in vitro* antioxidant activity of both extracts was determined by the 2,2-diphenyl-1-picrylhydrazyl (DPPH) assay. The biosynthesized CuO NPs from the *Bistorta amplexicaulis* extract showed anti-arthritic activity due to the presence of flavonoids and phenols, which showed a pain reliever effect by blocking the cyclo-oxygenase enzyme and has immune suppressant activity, thus securing the joint from destruction. The nanoparticles were characterized by zeta size, zeta potential, scanning electron microscopy (SEM), and FTIR spectroscopy. Forty-eight albino rats were divided randomly into six treatment groups.

**Results and Disscussion:**

The zeta size and zeta potential of the nanoparticles were 186.8 nm and −9.23 mV, respectively. Joint stiffness, spleen weight, thymus weight, and paw thickness showed a significant decrease after treatment with NPs. The hematological parameters such as red blood cells (RBCs) and hemoglobin showed a significant increase, while platelets and white blood cells (WBCs) showed a significant decrease in NP-treated groups. C-reactive protein (CRP), rheumatoid factor (RF), liver and kidney function biomarkers, tumor necrosis factor-α (TNF-α), and interleukin-6 (IL-6) levels showed a significant decrease at both low and high doses of green-synthesized CuO nanoparticles from the *Bistorta amplexicaulis* root extract. The final data were analyzed by one way and two-way analysis of variance (ANOVA) and Tukey’s multi-comparison test.

**Conclusion:**

So, from this study, it was concluded that both the plant root extract and green-synthesized CuO nanoparticles have anti-arthritic potential, but CuO NPs showed remarkable results.

## 1 Introduction

Rheumatoid arthritis (RA) is the most common kind of autoimmune arthritis (Merola et al., 2018). It is a chronic autoimmune condition marked by intense joint inflammation, which eventually damages the bone along with the cartilage of joints (Calabresi et al., 2018). In the United Kingdom, 1% of the population is affected by RA, and it has an effect on both the life expectancy and quality of life (Uhlig and Kvien, 2005).

The clinical presentation of rheumatoid arthritis involves pain, swelling, and nodule formation in joints. As the disease progresses, swan neck, boutonniere, and Z deformity of thumb can also occur. Inflammation of the synovium (called synovitis), bone and cartilage erosion, and angiogenesis also occur.

The reason for rheumatoid arthritis is not clearly known, but some researchers believe that epigenetic modifications, smoking, gender, and certain bacterial species such as *Porphyromonas gingivalis,* which also causes gingivitis, lead to RA (Catrina et al., 2016). The abovementioned reasons, as well as synovial injury of joints, hyperplasia, and infection releasing cytokines and inflammation, also lead to the modification of auto-antigens, i.e., damaging self-cells by considering them non-self.

Different types of cells and substances within the joint area mediate this immune response (i.e., chemokines and cytokines). There is no specified test for the detection of rheumatoid arthritis, but the erythrocyte sedimentation rate, C-reactive proteins, and presence of the RA factor can help in its diagnosis (Heidari, 2011; Van Steenbergen et al., 2018).

Synovial inflammation is caused by a combination of events, including immune cell migration to the site of inflammation and a failure of inflammatory cellular death. Leukocyte recruitment mechanisms in the synovial vasculature allow T cells to invade the inflammatory area. Rolling, adhesion, and transmigration events must be coordinated for the leukocyte adhesion cascade to occur. Leukocytes specifically travel along the endothelium, become activated, get attached to endothelial cells, and then move to the target location. This migration of T cells may be enhanced in RA, which would lead to a greater invasion of the stratum synovium by pro-inflammatory cytokines (Mellado et al., 2015).

Rheumatoid factors (RFs) such as the anti-citrullinated protein antibody (ACPA) of immunoglobulin G (IgG) and immunoglobulin M (IgM) cause the secretion of cytokines through macrophages such as tumor necrosis factor-alpha (TNF-α), interleukin-1 (IL-1), and interleukin-6 (IL-6) (Smolen et al., 2016). They also result in the stimulation of fibroblasts, which assist in rank-L expression. Osteoclasts, which are bone-specific cells, cause bone erosion in the advanced stages of the disease. These fibroblasts also cause the secretion of proteases that cause cartilage to breakdown, which results in cartilage degradation. These fibroblasts can travel from joint to joint as in the case of RA, from one side of the hand to the other (also called symmetrical arthritis) (Chiu and Ritchlin, 2017). If this inflamed pannus production increases further, it results in the irreversible damage of the joints.

Currently, no drug can completely cure rheumatoid arthritis, but certain agents are available that can decrease the severity and progression of RA. Disease-modifying anti-rheumatic drugs are used primarily for treating RA. These drugs have severe adverse effects on the body, including peptic ulcer, gastroesophageal reflux disease (GERD), nephrotoxicity, cardiovascular complications, and hematological toxicity (Katchamart et al., 2010).

The objective of conventional and alternative therapy is to reduce pain and stiffness in the joints with fewer pharmacological adverse effects. *Polygonum amplexicaule* D. Don has a long history of usage for treating pain, fractures, cardiovascular and cerebrovascular illnesses, and other conditions. Formerly known as *Polygonum amplexicaule*, *Bistorta amplexicaulis*, commonly known as Anjbar in Pakistan, is renowned for its antioxidant, antibacterial, anticancer, antifungal, and cardioprotective properties. It is also used frequently in the treatment of mastitis (Dilshad et al., 2010). Additionally, it inhibits atherosclerosis and promotes osteoblastic cell growth *in vitro* (Xiang et al., 2011).

Both micro and nano-systems can increase the effectiveness of therapeutic interventions in many ways. They can quickly identify the disease and retort to disease conditions straightaway by acting at the site by refining the patient’s compliance (Janakiraman et al., 2018). Nanotechnology is a recent field of study that focuses on the synthesis, biomanipulation, and use of nanoscale/nano-complex pharmaceuticals for various disease treatments and clinical investigations. Nanoparticles can be synthesized through various methods. The physical and chemical method for producing nanoparticles is expensive and may also result in the production of toxicants. Their biological synthesis is cheap, easy to produce, and minimizes the chemical load on the atmosphere (Kumari et al., 2019).

Freund’s complete adjuvant (FCA) is a blend of mycobacteria, non-metabolizable mineral oil, and surfactants (Heilborn et al., 2007). FCA is assumed to be the most active adjuvant to increase immunological responses for an antigen (Stils, 2005). Arthritis induced by the sub-dermal injection of FCA in mice provides a valuable model of nociceptive behavior induced by inflammation (Chillingworth and Donaldson, 2003). The objective of this study was to determine the anti-arthritic potential of green-synthesized copper oxide nanoparticles (CuO NPs) using the *B. amplexicaulis* root extract as a reducing agent.

## 2 Materials and methods

### 2.1 Chemicals and test kits

All chemicals were acquired from Sigma-Aldrich, United States; Freund’s complete adjuvant from Wuhan Zokeyo Biotechnology, China; hydroxychloroquine from Macter International Limited, Karachi; and copper sulfate from Riedel-de Haen Chemicalien GmbH, Germany. All test kits used during the present study were acquired from QCA, Spain.

### 2.2 Plant collection and extract preparation

*B. amplexicaulis* was collected from Ayubia National Park, Murree, and was identified by Dr. Mansoor Hameed, Associate Professor at the University of Agriculture, Faisalabad, with herbarium sheet no. 1117-22. After collection, the roots were washed with distilled water three times and shade-dried. After 2 weeks, the roots were grinded to coarse powder using a grinder. The extract was prepared by the maceration method. In this method of extraction of roots, 10 mg of powdered roots was mixed in 100 mL of hydroalcoholic solution (70:30). Then, it was stirred continuously and left for 72 h at room temperature under occasional stirring. After 3 days, the mixture was filtered using Whatman’s filter paper no. 1. After filtration, the extract was stored in an airtight container in a refrigerator at 4°C (Zhang et al., 2020).

### 2.3 Phytochemical testing

The extract was tested for the presence of different phytochemicals such as phenols and flavonoids using the ferric chloride test and saponins using the foam test. These phytochemicals have been used in the management of different diseases such as pain and inflammation. These phytochemicals can form strong bonds with metal ions and can be used in the synthesis of nanoparticles (Faisal et al., 2022).

### 2.4 Proximate composition analysis

Raw powdered plant material was used to assess physicochemical properties such as dry weight, moisture content, total carbohydrate, crude protein, crude fat, and total ash to determine nutritive values according to the Association of Official Analytical Chemists procedure (Khan et al., 2010). The given formula was used to obtain the total amount of carbohydrates from the percentages of crude fiber, protein, lipids, and ash determined above:

$$\text{Total carbohydrate }\left(\%\right)=100\;–\;\left[\text{moisture content }\left(\%\right)+\;\text{Crude fat }\left(\%\right)+\text{Crude Protein }\left(\%\right)+\;\text{Crude fiber }\left(\%\right)+\text{Total ash }\left(\%\right)\right],$$

$$\text{Nutritive value}=\left(\text{Fats percentage}\times 9\right)+\left(\text{carbohydrate percentage}\times 4\right)+\left(\text{protein percentage}\times 4\right).$$

Meanwhile, the concentration of elements in the prepared samples was determined using an atomic absorption spectrophotometer (Hitachi Polarized Zeeman AAS, Z-8200, Japan), following the conditions described in AOAC (1990).

### 2.5 Total flavonoid content

**FIGURE 1:**
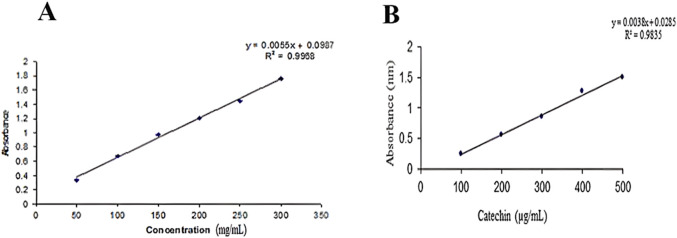
**(A)** Standard curve of gallic acid. **(B)** Standard curve of catechin.

The total flavonoid content of the plant was determined using a calibration curve of catechin (R^2^ = 0.9835). The total flavonoid and phenolic content was measured by the Folin and Ciocalteu method. All readings were taken in triplicate and averaged (Figures 1A, B).

### 2.6 Quantitative phytochemical analysis: reversed-phase high-performance liquid chromatography

A modified method suggested by Seal (2016) was adopted to analyze various bioactive compounds present in herbs.

### 2.7 Fourier-transform infrared spectroscopy

The *B. amplexicaulis* extract was subjected to FTIR analysis to detect different functional groups using an FTIR spectrometer (Spectrum Two™, PerkinElmer), accompanied with a detector (deuterated triglycine sulfate [DTGS]), germanium as a beam splitter, and connected to a window-based system (Spectrum, version 10.5.3) to identify different functional groups present in compounds.

### 2.8 2,2-Diphenyl-1-picrylhydrazyl assay

The ability of the plant extract to scavenge 2,2-diphenyl-1-picrylhydrazyl (DPPH) was assessed using a modified technique previously described (Ilavarasan et al., 2006). DPPH solution (0.002%) was freshly prepared in methanol with ascorbic acid as a standard solution. A measure of 2 mL of various concentrations of the plant extract and standard was combined with approximately 0.5 mL of the DPPH solution. After 15 min at room temperature, the absorbance at 517 nm was measured. The same reagent amounts, excluding the plant extract, were used to produce a blank solution. The formula was used to compute the percentage of inhibition after measuring absorbencies in triplicate.

### 2.9 Synthesis of CuO nanoparticles

**FIGURE 2:**
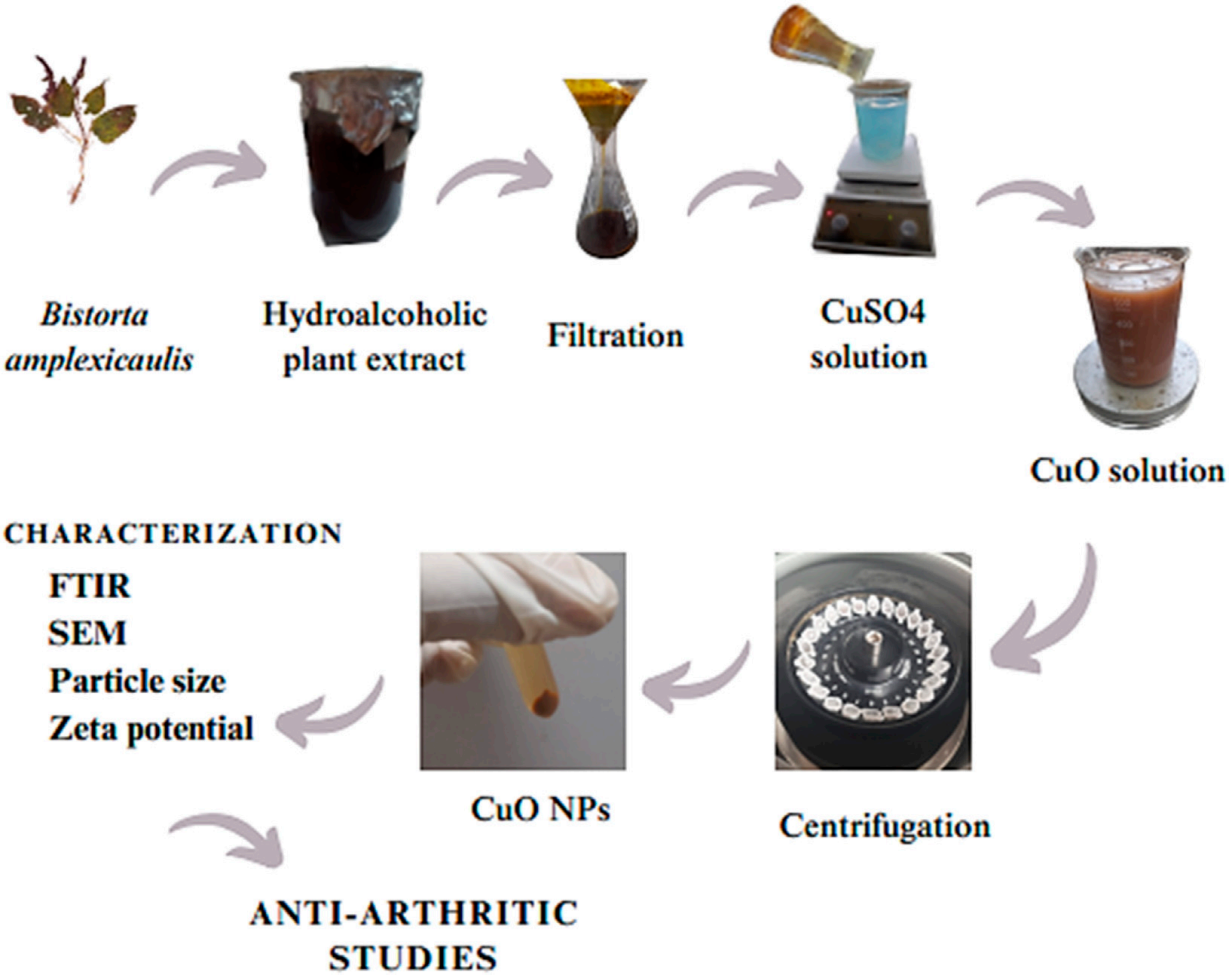
Step-by-step flowchart of the synthesis of CuO nanoparticles.

A co-precipitation method was used for the synthesis of CuO NPs. A measure of 5 mM copper sulfate solution was prepared by adding 0.79 g of copper sulfate to 1,000 mL distilled water under continuous stirring. A measure of 100 mL of the copper sulfate solution was added to a beaker and placed on a magnetic stirrer. Then, approximately 10 mL of the hydroalcoholic plant extract was added to the above solution and stirred at 60 rpm at 60°C for approximately 1 h. The solution was centrifuged at 6,000 rpm for approximately 5 min at 6°C. The supernatant was discarded, and pellets were washed with distilled water three times. The pellets were poured into a Petri dish and dried in a hot air oven at 85°C for 3 h. Then, the dried pellets were ground to fine powder and stored in an airtight container. A shift in color from light blue to dark gray indicated the formation of CuO NPs Figure 2.

### 2.10 Characterization of nanoparticles

Copper oxide nanoparticles were characterized by FTIR spectroscopy, scanning electron microscopy (SEM), and particle size and zeta potential analysis.

### 2.11 FTIR spectroscopy of CuO NPs

Copper oxide nanoparticles were subjected to FTIR analysis using an FTIR spectrophotometer (Spectrum Two™, PerkinElmer) accompanied with a detector (deuterated triglycine sulfate), a beam splitter, and a window-based system to detect different functional groups in the sample. For this purpose, 10 mg of the lyophilized sample was encapsulated in 100 mg of KBr pellet, and the sample was loaded in an FTIR spectroscope with a range of 4,000–500 cm^−1^.

### 2.12 SEM analysis

A measure of 50 mg of green-synthesized copper oxide nanoparticles using *B. amplexicaulis* roots were subjected to SEM analysis from the nanomaterial laboratory, physics department, Government College University, Faisalabad, for the determination of surface topography.

### 2.13 Particle size and zeta potential

Approximately 1 mL of CuO nanoparticles synthesized using *B. amplexicaulis* roots were used for the measurement of their zeta size by the dynamic light scattering procedure (Mujokoro et al., 2020).

### 2.14 *In vivo* study design

**FIGURE 3:**
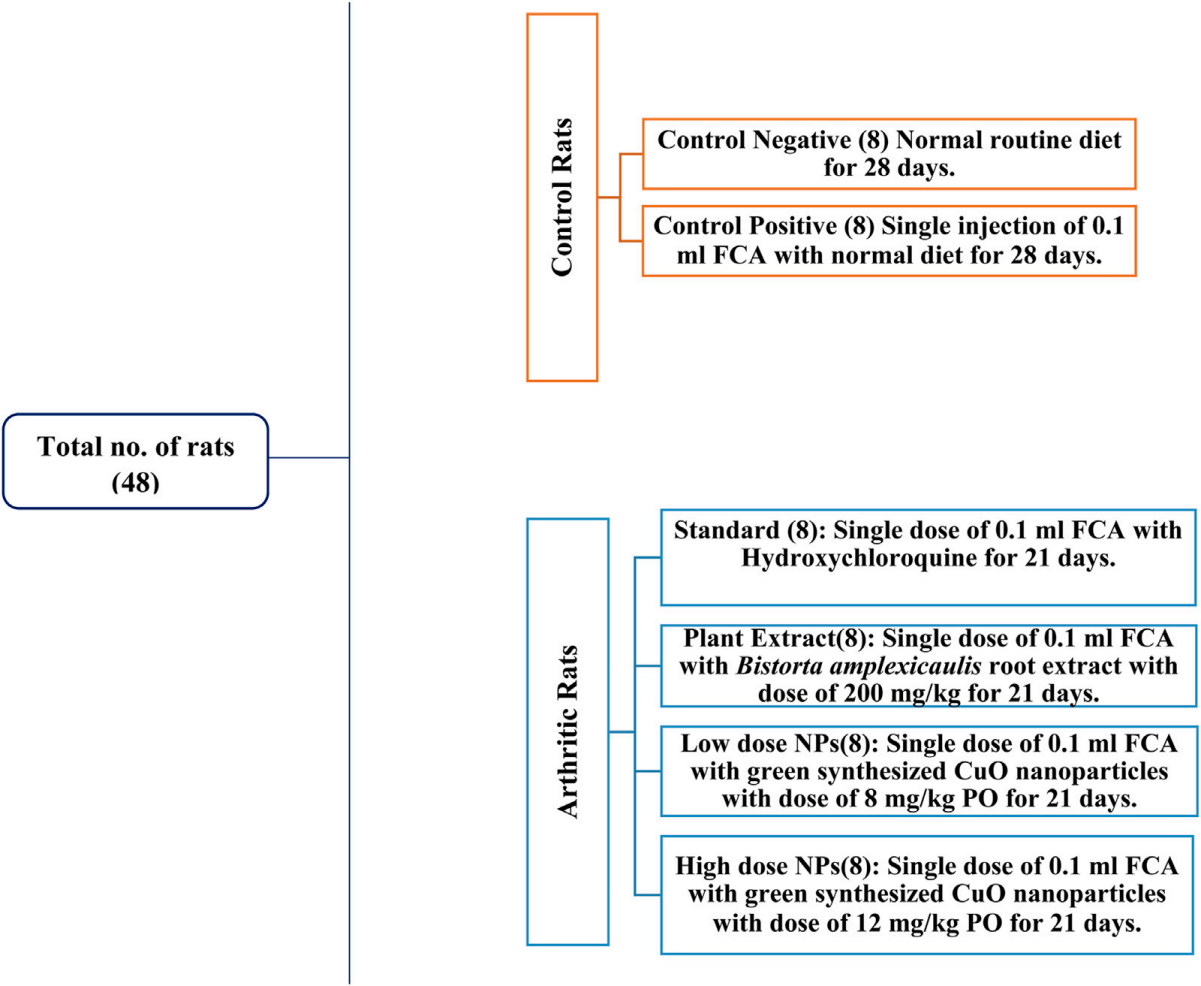
Study design of experimental rats.

Healthy albino Wistar rats of 6–8 weeks of age, weighing approximately 250–300 g, were used for this study. A total of 48 rats were used in this experimental trial and kept in cages in the animal house of the University of Agriculture, Faisalabad. Permission was sought from the Institutional Bioethical and Biosafety Committee (D. No. 1852/ORIC; dated: 30-03-2023) prior to the experiment. The rats were acclimatized for 1 week before the start of experiment. During this time, adequate food and water were provided to the rats (Figure 3).

### 2.15 Arthritis induction

Arthritis was induced by using Freund’s complete adjuvant. FCA was injected into the subplantar region of the left hind paw of rats at a dose of 0.1 mL. Induction took approximately 6–7 days to reach peak inflammation. The paw volume was measured using a Vernier caliper before and after the induction on every 0, 7, 14, 21, and 28^th^ day, as shown in the figure (Bhalekar et al., 2016). On day 7 after the induction, treatment was started with hydroxychloroquine in the standard group, a simple extract dose in treatment 1, a low-nanoparticle dose in treatment 2, and high nanoparticles in treatment 3. The treatment protocol was followed for a total of 21 days from day 7 to day 28. The weight of the rats was measured on days 0, 7, 14, 21, and 28 using a weighing balance to check the effect of the treatment. The paw thickness was measured using a Vernier caliper on days 0, 7, 14, 21, and 28 day in all the groups to check the effectiveness of the treatment.

### 2.16 Mobility score

The mobility score was determined on days 0, 7, 14, 21, and 28 in all the experimental groups for the estimation of the effectiveness of green-synthesized nanoparticles using *B. amplexicaulis* roots. The score was determined from 0 to 6 grade (Arora et al., 2015).

### 2.17 Joint stiffness

Joint stiffness was measured on every seventh day from day 7 to day 28 to check the effectiveness of the treatment. The grading system of 0–2 was followed (Arora et al., 2015).

### 2.18 Decapitation and *in vivo* testing

On day 29 of treatment, the animals were anesthetized using the drop method with 5% isoflurane. The animal to be anesthetized was placed in a transparent plastic glass anesthesia chamber of volume 3.5 L. Isoflurane was introduced into the chamber using a cotton swab soaked with 1.32 mL of isoflurane. The quantity of isoflurane required was calculated using the ideal gas law (PV = nRT, where P = pressure, V = volume, n = moles of isoflurane, R = universal gas constant (8.314), and T = temperature). The animal was left in the chamber for 5 min and then decapitated when fully sedated following the bioethics protocol. Blood samples of each rat from each group were collected in ethylenediaminetetraacetic acid (EDTA) tubes for analyzing the complete blood count (CBC).

The tests were performed in the Diagnostic Laboratory of the Department of Pathology, University of Agriculture, Faisalabad. Blood was collected in EDTA tubes and then centrifuged at 5,000 rpm for 5 min to collect the serum. The serum was collected in labeled Eppendorf tubes and stored in the ultra-freezer at −20°C for further testing of oxidative stress parameters, inflammatory mediators, and biochemical testing. After decapitation, organs including the spleen, thymus, liver, kidney, and paw were collected. The spleen and thymus were collected and weighed for the immune organ index (Chui et al., 2019). After weighing, they were discarded.

The liver, kidney, and paw were collected in 10% normal buffer formalin for histopathological studies. Serum biochemical parameters such as the liver function and kidney function and cytokine biomarkers including TNF-α, IL-6, C-reactive protein (CRP), and RA factor levels were determined. On day 29, FCA-induced arthritic rats were subjected to radiological examination (exposure was 50 KVp and 200 mAs) using an X-ray unit (KXO-12R, Toshiba, Japan). Qualitative assessment was performed for the swelling of soft tissues, narrowing of joint space, joint ankylosis, osteolysis, and periosteal reaction at the metatarsal area (Ananthi et al., 2017).

### 2.19 Statistical analysis

Statistical analysis was done by one-way and two-way ANOVA and Tukey’s multi-comparison test using GraphPad Prism software (Murtaza et al., 2021).

## 3 Results

### 3.1 Proximate composition analysis

Parameters including the dry weight, moisture content, crude protein, fats, carbohydrates, fiber, and total ash were determined for the estimation of the nutritive value. Moisture content was found to be 4.7%; dry matter, 95%; crude fiber, 8%; crude protein, 10.93%; carbohydrates, 63.59%; crude fat, 5%; and total ash, 7.78%. The nutritive value was found to be 343.04 kJ/mol.

### 3.2 Mineral analysis

The concentration of various minerals including manganese, cobalt, iron, calcium, lead, copper, zinc, and cadmium was determined in *B. amplexicaulis* roots using the AAS. The amount of minerals was found to be 0.034; Co, 0.2035; Fe, 0.3162; Ca, 3.45; Pb, 0.0727; Cu, 0.1; Cd, 0.0327; and Zn, 0.08 ppm.

### 3.3 Qualitative phytochemical analysis of the extract

The *B. amplexicaulis* extract was phytochemically analyzed for the presence of bioactive compounds in the plant. The results showed the presence of acids, cardiac glycosides, flavonoids, fixed oils, phenols, amino acids, betacyanins, phlobatannins, alkaloids, quinones, tannins, saponins, volatile oils, and terpenoids, while no gums were detected in the sample.

### 3.4 Total phenolic and flavonoid content

The total phenolic content was estimated to be 585.847 ± 1.423 mg, and the total flavonoid content was found to be 274.98 ± 1.388 mg.

### 3.5 HPLC

**FIGURE 4:**
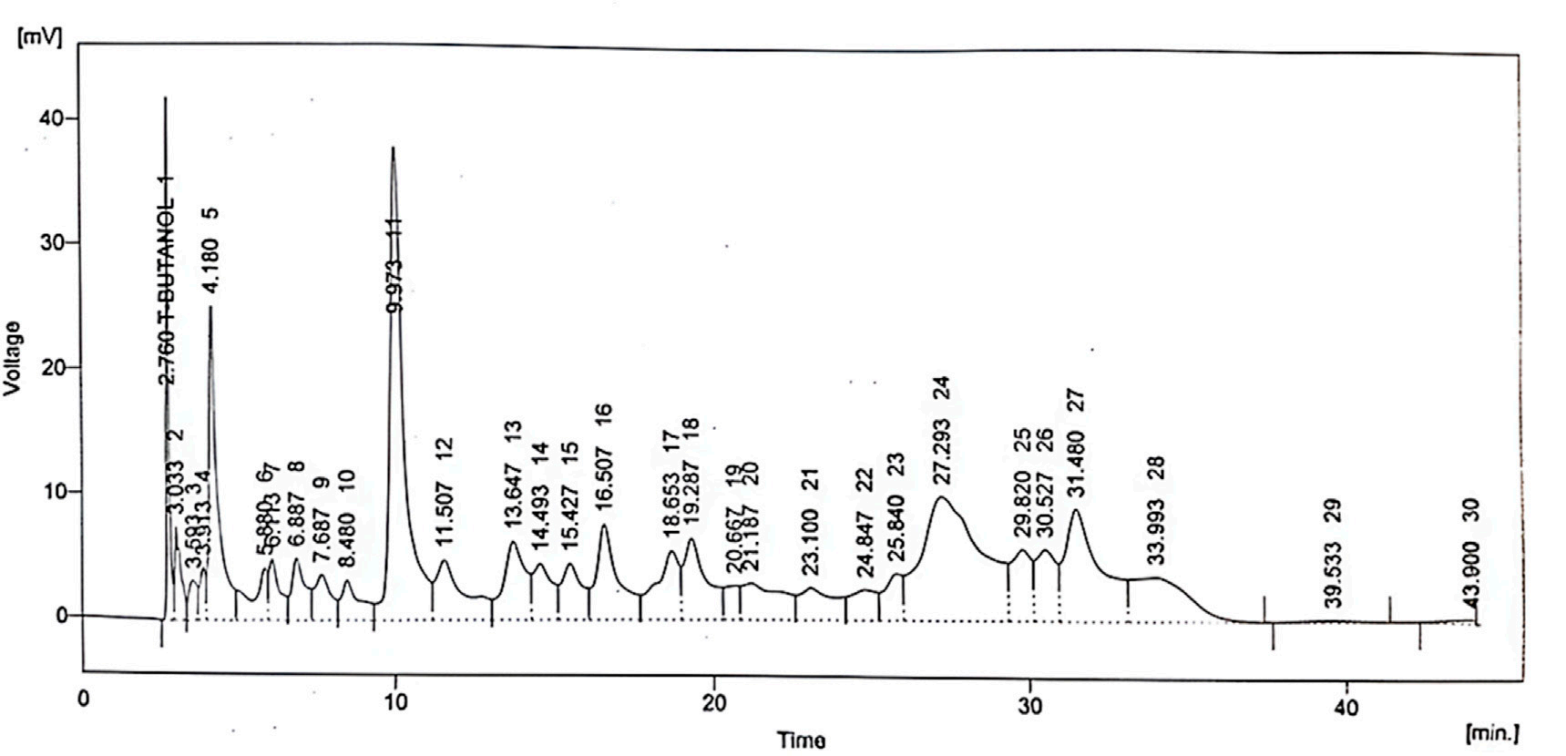
Chromatographic spectrum of *Bistorta amplexicaulis*.

Various phytochemical compounds including quercetin, gallic acid, vanillic acid, benzoic acid, chlorogenic acid, syringic acid, m-coumaric acid, and cinnamic acid were identified via the HPLC technique in the *B. amplexicaulis* extract, and their retention time and respective concentrations are given in Table 1 and Figure 4.

**TABLE 1:** Concentrations and retention times of phytocompounds detected in *B. amplexicaulis*

Sr. no.	Compound	Retention time	Area mV. s	Area %	Concentration, ppm
1	Quercetin	2.760	221.607	2.7	11.74
2	Gallic acid	4.180	481.424	5.8	17.33
3	Vanillic acid	13.647	289.658	3.5	17.95
4	Benzoic acid	14.493	184.074	2.2	19.51
5	Chlorogenic acid	15.427	195.064	2.4	15.21
6	Syringic acid	16.507	367.493	4.4	9.18
7	m-Coumaric acid	20.667	87.704	1.1	1.05
8	Cinnamic acid	24.847	144.621	1.7	5.06

### 3.6 FTIR spectroscopy

**FIGURE 5:**
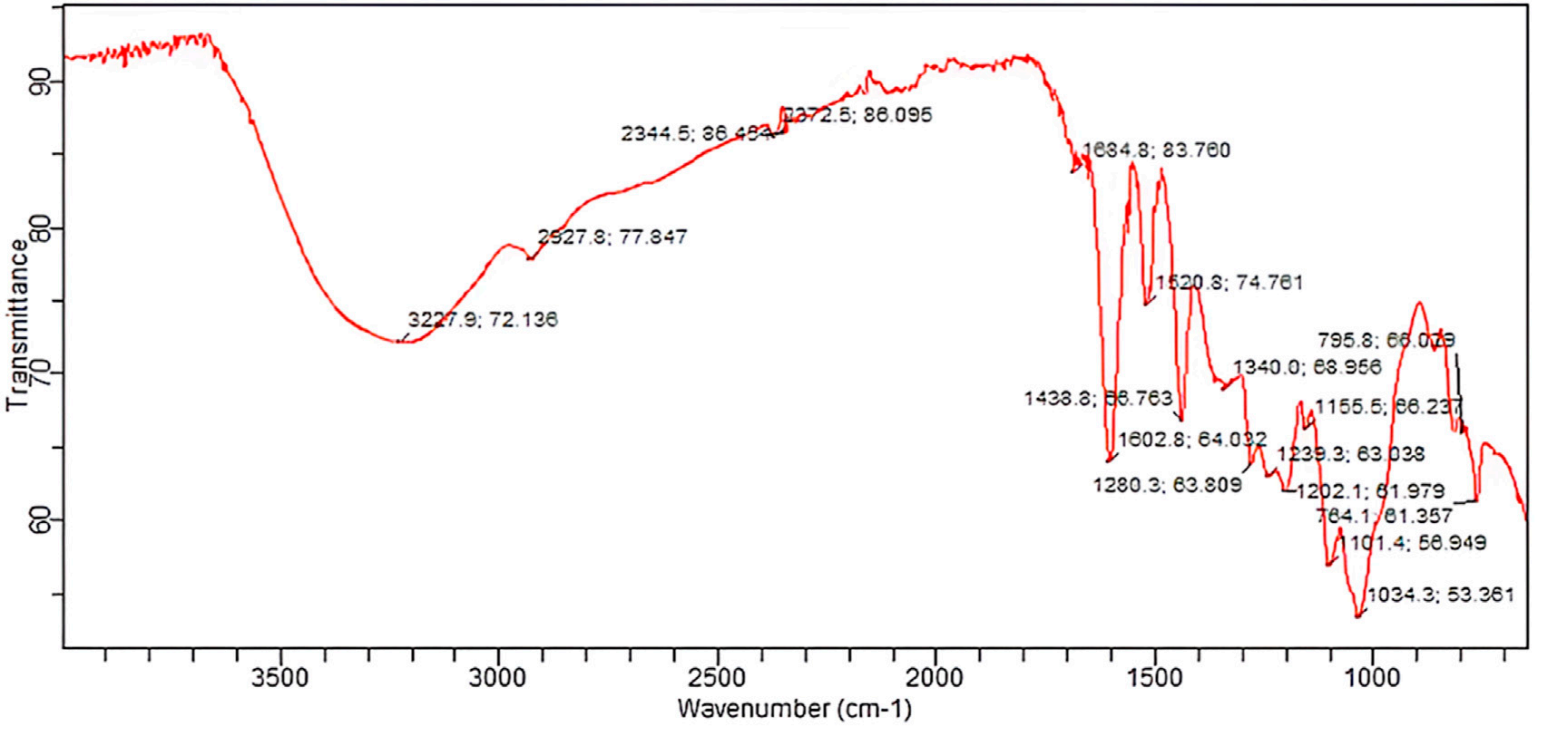
FTIR spectrum of the *B. amplexicaulis* extract.

The possible role of the *B. amplexicaulis* root extract was determined by FTIR spectroscopy. The FTIR analysis showed the presence of different functional groups at different peaks, including an O-H bond at 3,227.9 cm^−1^, C-H bending at 1,684.8 cm^−1^, C-C bending at 1,438.8 cm^−1^, CF stretching at 1,280.3 cm^-1^, N-H bending at 1,620.8 cm^−1^, C=C stretching at 1,602.8 cm^−1^, O-H bending at 1,340.0 cm^−1^, C-O stretching at 1,239.3 cm^−1^, C-F stretching at 1,155.5 cm^−1^, C-Cl stretching at 795.8 cm^−1^, and C-N stretching at 1,101.4 cm^−1^, as shown in Figure 5.

### 3.7 DPPH% inhibition

The antioxidant potential of the *B. amplexicaulis* extract was determined through the DPPH assay *in vitro*. The extract exhibited dose–response antioxidant activity in the DPPH radical assay compared to a standard (ascorbic acid), and its percentage inhibitions calculated from the results are given in Table 2.

**TABLE 2:** Percentage inhibition of *B. amplexicaulis* and ascorbic acid for the DPPH assay.

Concentration	% inhibition	
(µg/mL)	*B. amplexicaulis*	Ascorbic acid
100	25.64	83.76
200	37.78	86.27
300	52.43	87.58
400	67.21	89.03

### 3.8 Characterization of CuO NPs

#### 3.8.1 FTIR spectroscopy

The copper oxide nanoparticles were subjected to FTIR analysis using an FTIR spectrophotometer (Spectrum Two™, PerkinElmer) accompanied with a detector (deuterated triglycine sulfate), a beam splitter, and a window-based system to detect different functional groups in the sample. For this purpose, 10 mg of the lyophilized sample was encapsulated in 100 mg of KBr pellet, and the sample was loaded in an FTIR spectroscope with a range of 4,000–500 cm^−1^.

**FIGURE 6:**
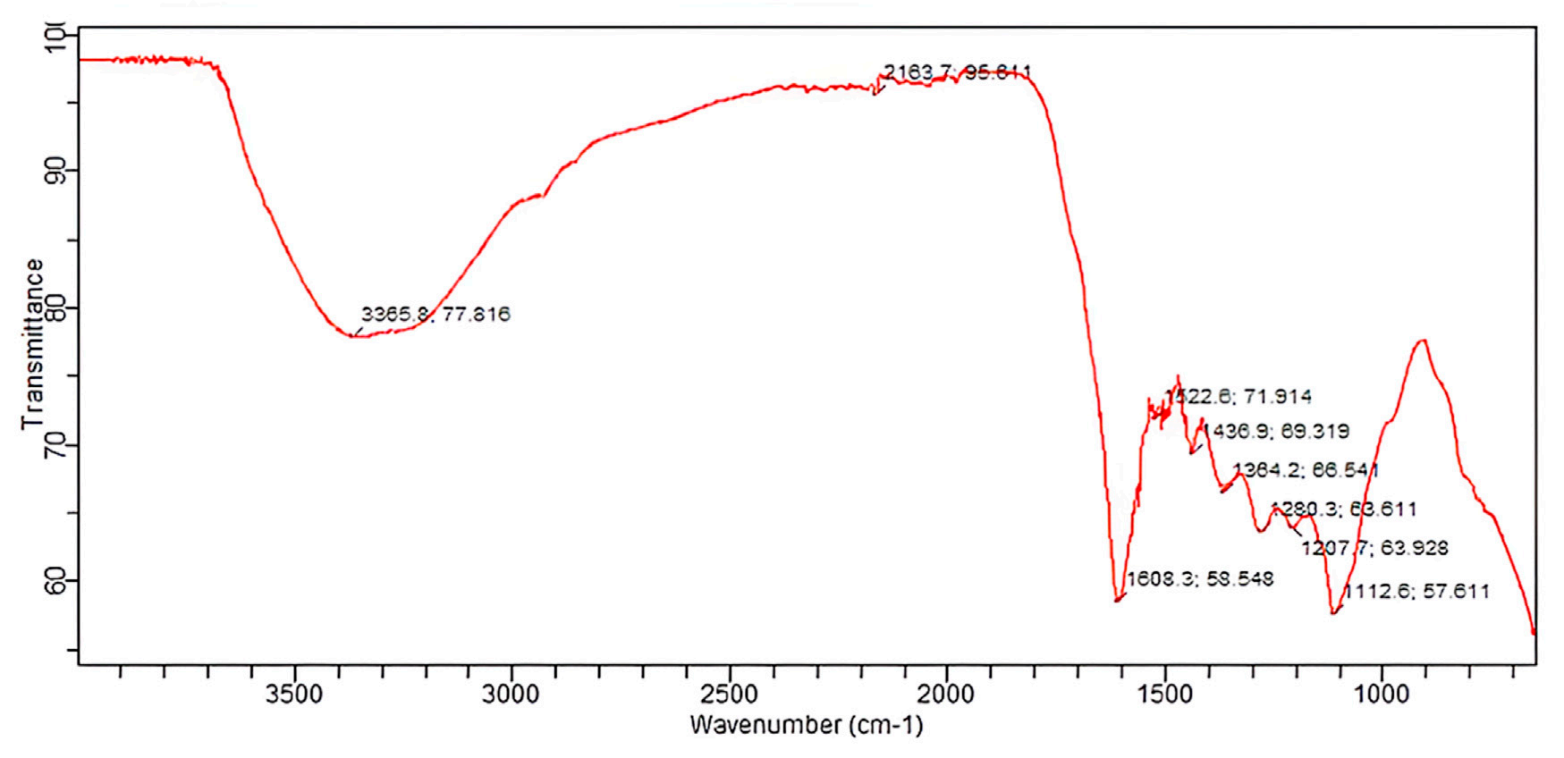
FTIR spectrum of CuO NPs.

The FTIR analysis of *B. amplexicaulis* showed the presence of different functional groups at different frequencies, including O-H at 3,365.8 cm^-1^, O-H at 2,163.7 cm^−1^, C=O at 1,608.3 cm^−1^, N-O at 1,522.6 cm^−1^, C-C at 1,436.9 cm^−1^, O-H at 1,364.2 cm^−1^, CN at 1,280.3 cm^−1^, C-O at 1,207.7 cm^−1^, and C-N at 1,112.6 cm^−1^, as shown in Figure 6.

The comparison of the FTIR spectra of the plant extract and CuO NPs showed a similarity in the peaks at wavelengths of O-H bonds at 3,227.9 cm^−1^ and 3,365.8 cm^−1^, C-C bending at 1,438.8 cm^−1^ and C-C at 1,436.9 cm^−1^, O-H bending at 1,340.0 cm^−1^ and O-H at 1,364.2 cm^−1^, C-O stretching at 1,239.3 cm^−1^ and C-O at 1,207.7 cm^−1^, and C-N stretching at 1,101.4 cm^−1^ and C-N at 1,112.6 cm^−1^, indicating the presence of these functional groups in both samples, and a slight shift in the peaks indicates the involvement of the plant extract in the synthesis of CuO NPs.

#### 3.8.2 SEM analysis

**FIGURE 7:**
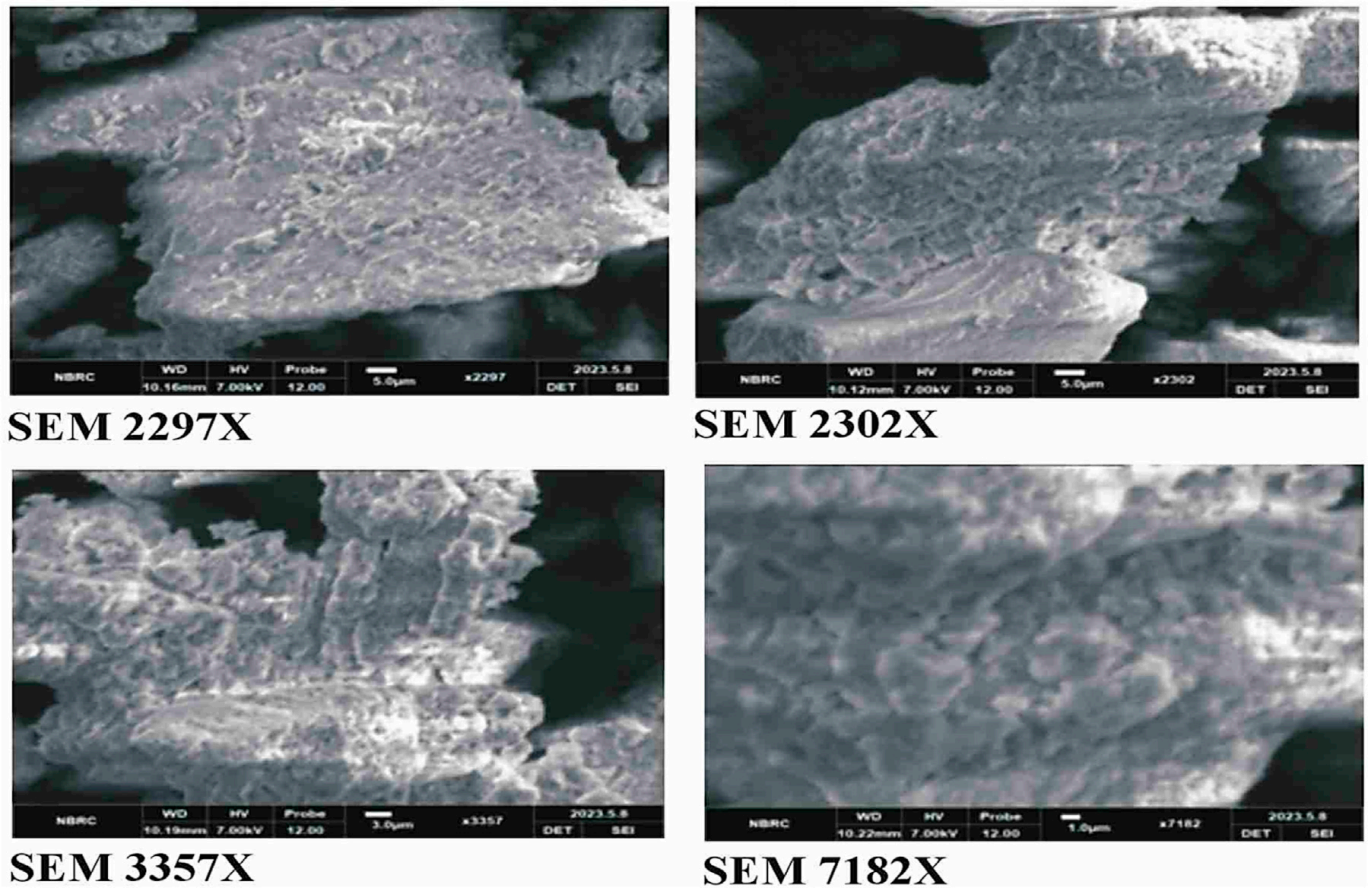
SEM analysis of green synthesized CuO nanoparticles from the *B. amplexicaulis* root extract.

The SEM analysis of CuO nanoparticles indicated that the particles were amorphous in shape and in cluster form. The surface of nanoparticles was porous in nature, according to the analysis (Figure 7).

#### 3.8.3 Particle size

The zeta size results revealed the size of nanoparticles to be 186.8 nm.

#### 3.8.4 Zeta potential

**FIGURE 8:**
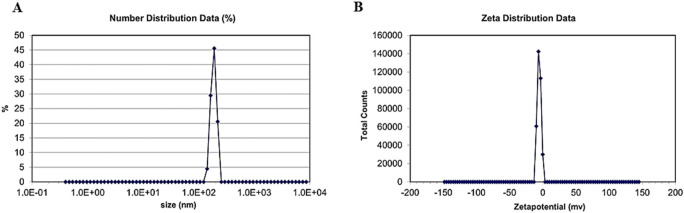
**(A)** Distribution of zeta size. **(B)** Zeta potential of green synthesized CuO nanoparticles from the *B. amplexicaulis* root extract.

The results showed that the potential charge present on the surface of biosynthesized CuO nanoparticles is −9.23 mV, which means that nanoparticles are strongly anionic, as shown in Figure 8.

#### 3.8.5 Anti-arthritic activity in adjuvant-induced arthritis in albino Wistar rats

##### 3.8.5.1 Effect of the green synthesized CuO nanoparticles from the *B. amplexicaulis* root extract on body weight (g)

**FIGURE 9:**
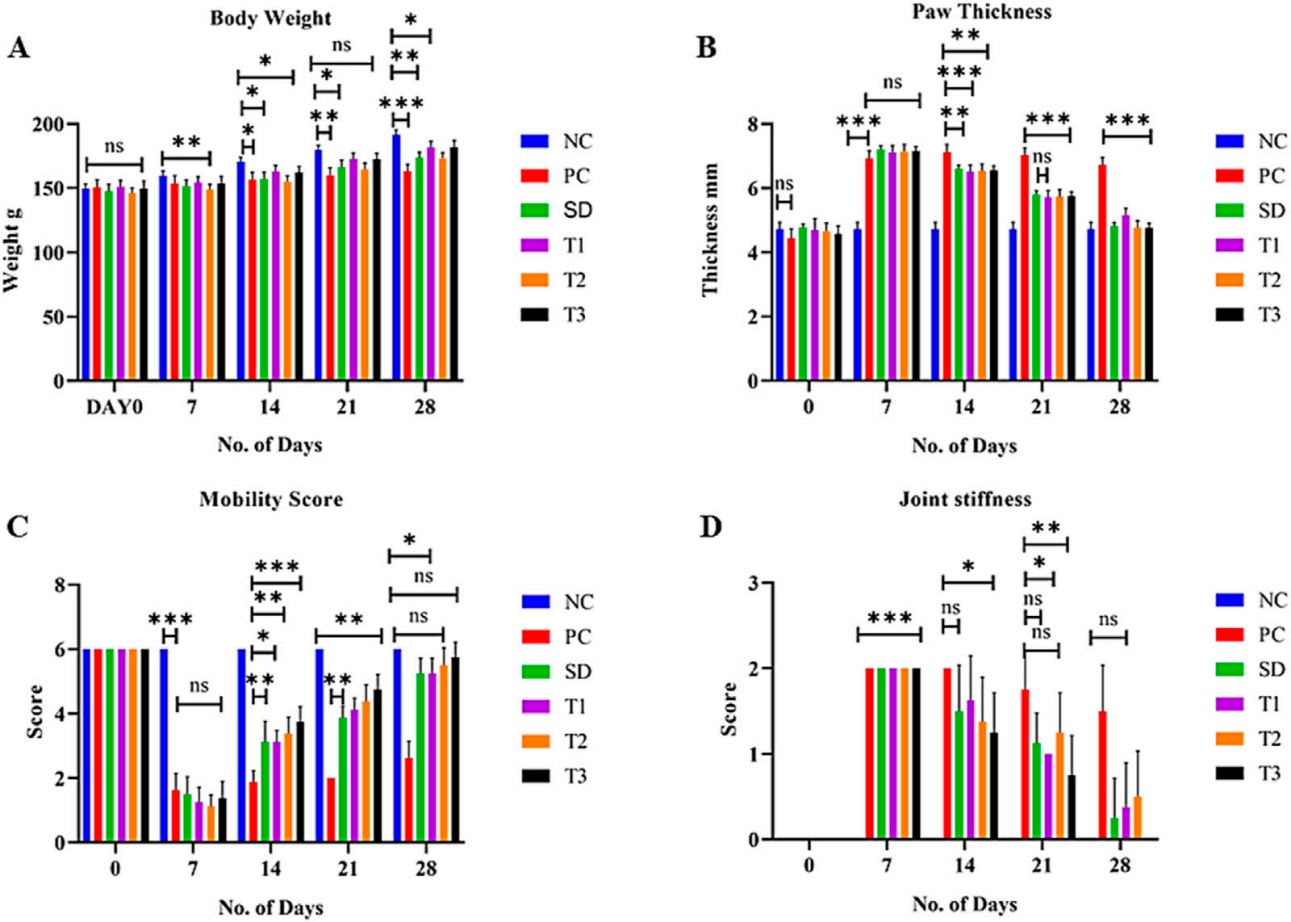
Effect of green synthesized CuO nanoparticles of the *B. amplexicaulis* root extract on **(A)** body weight; **(B)** paw thickness; **(C)** mobility score; and **(D)** joint stiffness score. Control negative (NC), control positive (PC), standard dose (SD), plant extract (T1), low-dose nanoparticles (T2), and high-dose nanoparticles (T3). Significance value: *p < 0.05, **p < 0.001, and ***p < 0.0001. Comparison of the disease group with other groups.

The body weight of all experimental animals was measured on days 0, 7, 14, 21, and 28 of the experiment. After the induction of arthritis, a significant (*p* < 0.01) increase in weight was exhibited by treatment groups, but a gradual increase in weight was exhibited by the positive control group (Figure 9A).

##### 3.8.5.2 Effect of the green synthesized CuO nanoparticles from the *B. amplexicaulis* root extract on paw thickness

The paw thickness was significantly (*p* < 0.001) increased in all groups on day 7 compared to the negative control group after the induction of arthritis. The paw thickness began to reduce significantly (*p* < 0.001) after the initiation of treatment, as shown in Figure 9B.

##### 3.8.5.3 Effect of the green synthesized CuO nanoparticles from the *B. amplexicaulis* root extract on the mobility score

The statistical analysis of the mobility score showed a significant (*p* < 0.001) variation among different treatment groups on different days. A significant (*p* < 0.001) increase in the mobility score was observed in treatment groups compared to the positive control group. A nonsignificant variation was observed between low- and high-dose nanoparticles (Figure 9C).

##### 3.8.5.4 Effect of the green synthesized CuO nanoparticles from the *B. amplexicaulis* root extract on joint stiffness

The statistical analysis of joint stiffness showed a significant (*p* < 0.001) variation among different treatment groups on different days. A significant (*p* < 0.001) reduction in joint stiffness was observed in the standard group compared to the positive control group. A nonsignificant variation was observed between negative control and high-dose nanoparticles on day 28 of the treatment, as shown in Figure 9D.

##### 3.8.5.5 Effect of the green synthesized CuO nanoparticles from the *B. amplexicaulis* root extract on the spleen organ index

**FIGURE 10:**
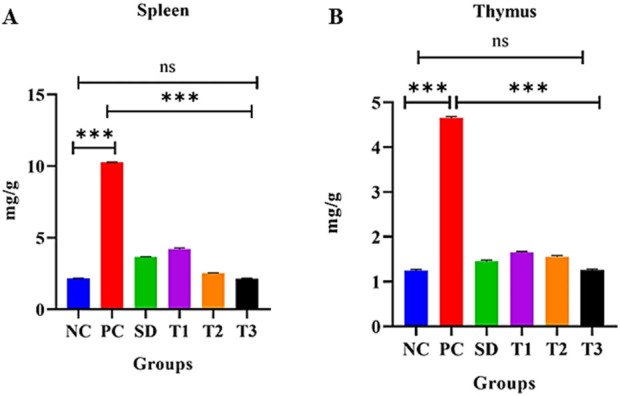
Effect of green synthesized CuO nanoparticles of the *B. amplexicaulis* root extract on **(A)** spleen weight index and **(B)** thymus weight index of different groups of rats: negative control (NC), positive control (PC), standard dose (SD), plant extract (T1), low-dose nanoparticles (T2), and high-dose nanoparticles (T3). Significance value: *p < 0.05, **p < 0.001, and ***p < 0.0001. Comparison of the disease group with other groups.

The statistical analysis of the spleen weight index exhibited great variation among the treatment groups. The statistical analysis displayed a significant (*p* < 0.001) increase in the weight of the spleen in the arthritic control group compared to the control negative group. The negative control group displayed a nonsignificant association with the high-dose NP-treated group but showed a significant association with all the other groups. A significant reduction in the weight of the spleen was observed in the *B. amplexicaulis* extract and low- and high-dose-treated groups, as shown in Figure 10A.

##### 3.8.5.6 Effect of green synthesized CuO nanoparticles from the *B. amplexicaulis* root extract on the thymus organ index

The statistical assessment of the thymus weigh index exhibited a great variation among treatment groups. The association between the negative control and arthritic (positive) control was found to be significant (*p* < 0.001). Negative control and high-dose NPs were found to exhibit a nonsignificant association, while all other groups were highly significant. Moreover, a decrease in the weight of the thymus was observed in the simple extract dose, low-dose nanoparticles, and high-dose nanoparticles, as shown in Figure 10B.

##### 3.8.5.7 Effect of green synthesized CuO nanoparticles from the *B. amplexicaulis* root extract on red blood cells (10^12^/L)

**FIGURE 11:**
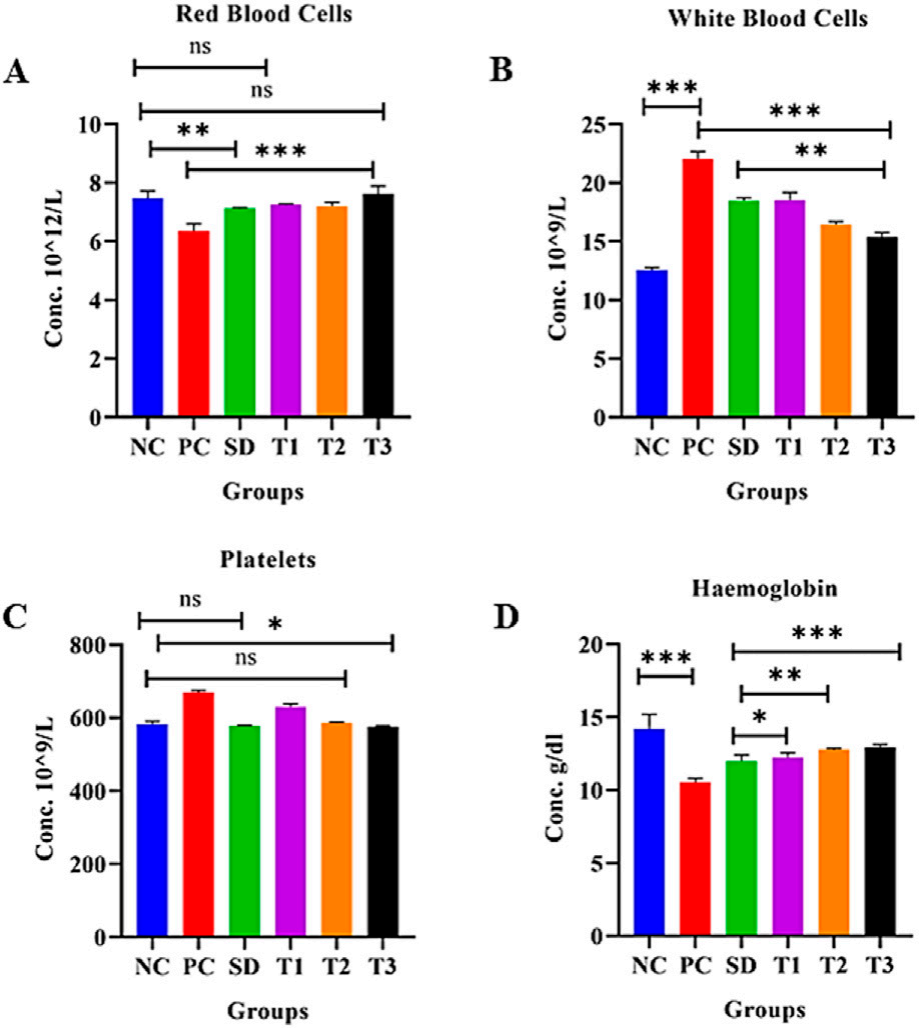
Effect of green synthesized CuO nanoparticles of the *B. amplexicaulis* root extract on **(A)** RBC, **(B)** WBC, **(C)** platelets, and **(D)** Hb. Negative control (NC), positive control (PC), standard dose (SD), plant extract (T1), low-dose nanoparticles (T2), and high-dose nanoparticles (T3). Significance value: *p <0.05, **p < 0.001, and ***p < 0.0001. Comparison of the disease group with other groups.

The statistical analysis of red blood cells (RBCs) showed a significant (*p* < 0.001) decrease in RBCs in the positive control group compared to the negative control. The negative control, low-dose NP-, and high-dose NP-treated groups were found to be nonsignificant, while other groups were significantly different from each other, as shown in Figure 11A, Table 3.

**TABLE 3:** Comparison of hematological parameters of different study groups.

Hematological parameter	Mean ± standard deviation of study groups					
	Negative control	Positive control	Standard drug	*B. amplexicaulis* extract	Low-dose CuO nanoparticles	High-dose CuO nanoparticles
RBCs	7.501 ± 0.260	6.386 ± 0.252	7.126 ± 0.033	7.259 ± 0.039	7.150 ± 0.036	7.499 ± 0.075
WBCs	12.568 ± 0.205	22.043 ± 0.626	18.486 ± 0.234	18.528 ± 0.653	16.434 ± 0.270	15.376 ± 0.375
Platelets	583.250 ± 7.005	670.375 ± 4.340	577.375 ± 1.923	630.500 ± 8.053	585.500 ± 2.878	575.500 ± 3.162
Hb	14.201 ± 0.970	10.525 ± 0.276	11.981 ± 0.407	12.238 ± 0.310	12.778 ± 0.080	12.921 ± 0.198

##### 3.8.5.8 Effect of green synthesized CuO nanoparticles from the *B. amplexicaulis* root extract on white blood cells (10^9^/L)

The statistical analysis of white blood cells (WBCs) showed a significant (*p* < 0.001) increase in WBC count in the arthritic control group compared to the negative control group. However, the treatments showed a significant reduction (*p* < 0.001) in the WBC level compared to the arthritic group, as shown in the table and figure: the graph confirmed a significant decrease in the level of WBCs in the standard, plant extract, low-dose, and high-dose NP-treated groups (Figure 11B) Table 3.

##### 3.8.5.9 Effect of green synthesized CuO nanoparticles from the *B. amplexicaulis* root extract on the platelet count (10^9^/µL)

In the statistical assessment of the platelet count in treatment groups, arthritic control exhibited a significant (*p* < 0.001) increase in the platelet count compared to the negative control. A nonsignificant association was found between the standard and low-dose nanoparticle-treated groups. Negative control and high-dose NPs were found to be less significantly associated. The number of platelets decreased to normal levels in all three treatment groups (Figure 11C) Table 3.

The statistical analysis of Hb showed a significant (*p* < 0.001) variation between the negative and positive control groups. A significant (*p* < 0.001) association was also observed between the positive control and other three treatment groups. A nonsignificant association was observed between the standard and simple extract group. All three treatment groups showed a significant increase in Hb levels, as shown in Figure 11D, Table 3.

##### 3.8.5.10 Effect of green synthesized CuO nanoparticles from the *B. amplexicaulis* root extract on alanine aminotransferase levels (U/L)

**FIGURE 12:**
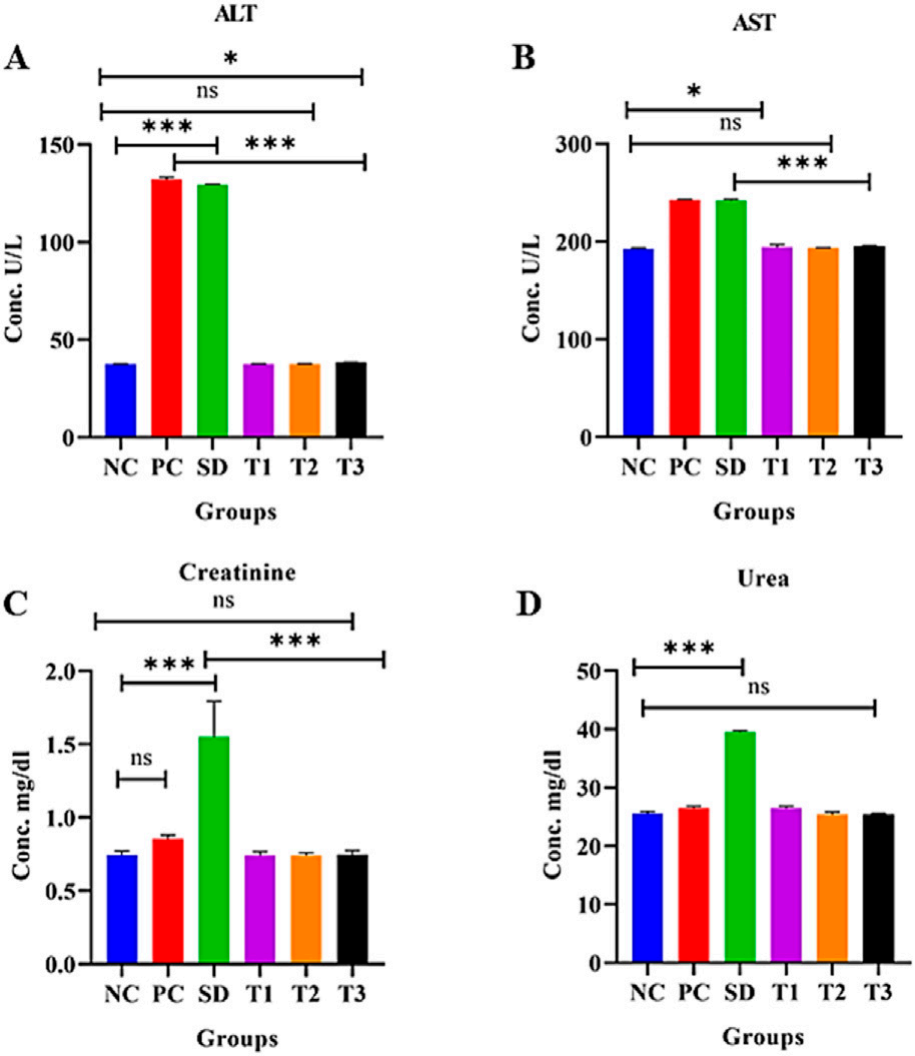
Effect of green synthesized CuO nanoparticles of the *B. amplexicaulis* root extract on **(A)** ALT, **(B)** AST, **(C)** creatinine, and **(D)** urea levels. Negative control (NC), positive control (PC), standard dose (SD), plant extract (T1), low-dose nanoparticles (T2), and high-dose nanoparticles (T3). Significance value: *p <0.05, **p < 0.001, and ***p < 0.0001. Comparison of the disease group with other groups.

The statistical analysis revealed a significant increase in ALT levels (*p* < 0.0001) in the positive control group compared to the negative control group. A significant association (*p* < 0.0001) was also observed between the standard treatment group and the negative control group. However, there was a nonsignificant relationship between the negative control group and treatment groups 1 and 2. Additionally, a marginally significant relationship was found between the negative control and treatment group 3, as shown in Figure 12A.

##### 3.8.5.11 Effect of green synthesized CuO nanoparticles from the *B. amplexicaulis* root extract on aspartate aminotransferase levels (U/L)

Statistical analysis of aspartate aminotransferase (AST) levels indicated a significant increase (*p* < 0.01) in the positive control group compared to the negative control group. Additionally, a significant increase (*p* < 0.001) in AST levels was observed in the standard treatment group relative to the negative control group. The analysis also revealed a nonsignificant association between the negative control group and treatment groups 1 and 2. Furthermore, a marginally nonsignificant association was found between the negative control and treatment group 3, as shown in Figure 12B.

##### 3.8.5.12 Effect of green synthesized CuO nanoparticles from the *B. amplexicaulis* root extract on serum creatinine levels (mg/dL)

The statistical analysis revealed a significant increase in creatinine levels (*p* < 0.0001) in the standard treatment group compared to the negative control group. However, no significant variation was observed between the negative control and the three other treatment groups. There was no difference in creatinine levels between the positive and negative control groups. Both the plant extract and nanoparticles showed a reduction in creatinine levels compared to the standard treatment group, as shown in Figure 12C.

##### 3.8.5.13 Effect of green synthesized CuO nanoparticles from the *B. amplexicaulis* root extract effect on serum urea levels (mg/dL)

Statistical analysis showed a significant increase in urea levels (*p* < 0.001) in the standard treatment group compared to the negative control group. However, there was no significant variation between the positive and negative control groups. Additionally, a nonsignificant association was observed between the negative control group and both the low- and high-dose NP treatment groups, as shown in Figure 12D.

##### 3.8.5.14 Effect of green synthesized CuO nanoparticles from the *B. amplexicaulis* root extract on serum CRP levels

**FIGURE 13:**
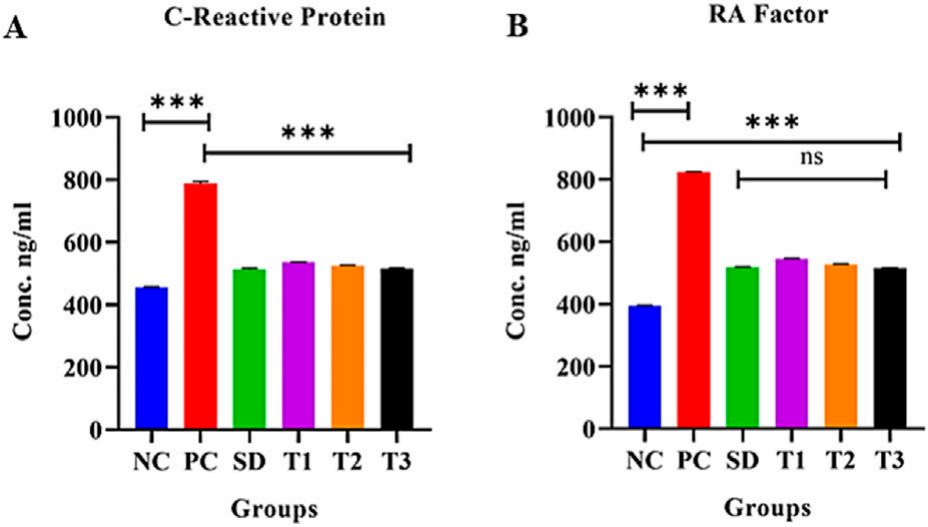
Effect of green synthesized CuO nanoparticles of the *B. amplexicaulis* root extract on **(A)** CRP and **(B)** RA factor. Negative control (NC), positive control (PC), standard dose (SD), plant extract (T1), low-dose nanoparticles (T2), and high-dose nanoparticles (T3). Significance value: *p <0.05, **p < 0.001, and ***p < 0.0001. Comparison of the disease group with other groups.

The statistical analysis revealed a significant increase in CRP levels (*p* < 0.0001) in the positive control group compared to the negative control group. In contrast, a significant decrease in CRP levels (*p* < 0.0001) was observed in the other treatment groups. Additionally, a significant association (*p* < 0.0001) was found between treatment 2 and treatment 3 groups, as shown in Figure 13A.

##### 3.8.5.15 Effect of green synthesized CuO nanoparticles from the *B. amplexicaulis* root extract on serum RF levels

The statistical analysis revealed a significant increase in RA factor levels (*p* < 0.001) in the positive control group compared to the negative control group. In contrast, a significant decrease in RA factor levels (*p* < 0.001) was observed in the other treatment groups. Additionally, the standard treatment group and treatment 3 group showed a nonsignificant association with each other, as shown in Figure 13B.

##### 3.8.5.16 Effect of green synthesized CuO nanoparticles from the *B. amplexicaulis* root extract effect on superoxide dismutase activity in liver tissue

**FIGURE 14:**
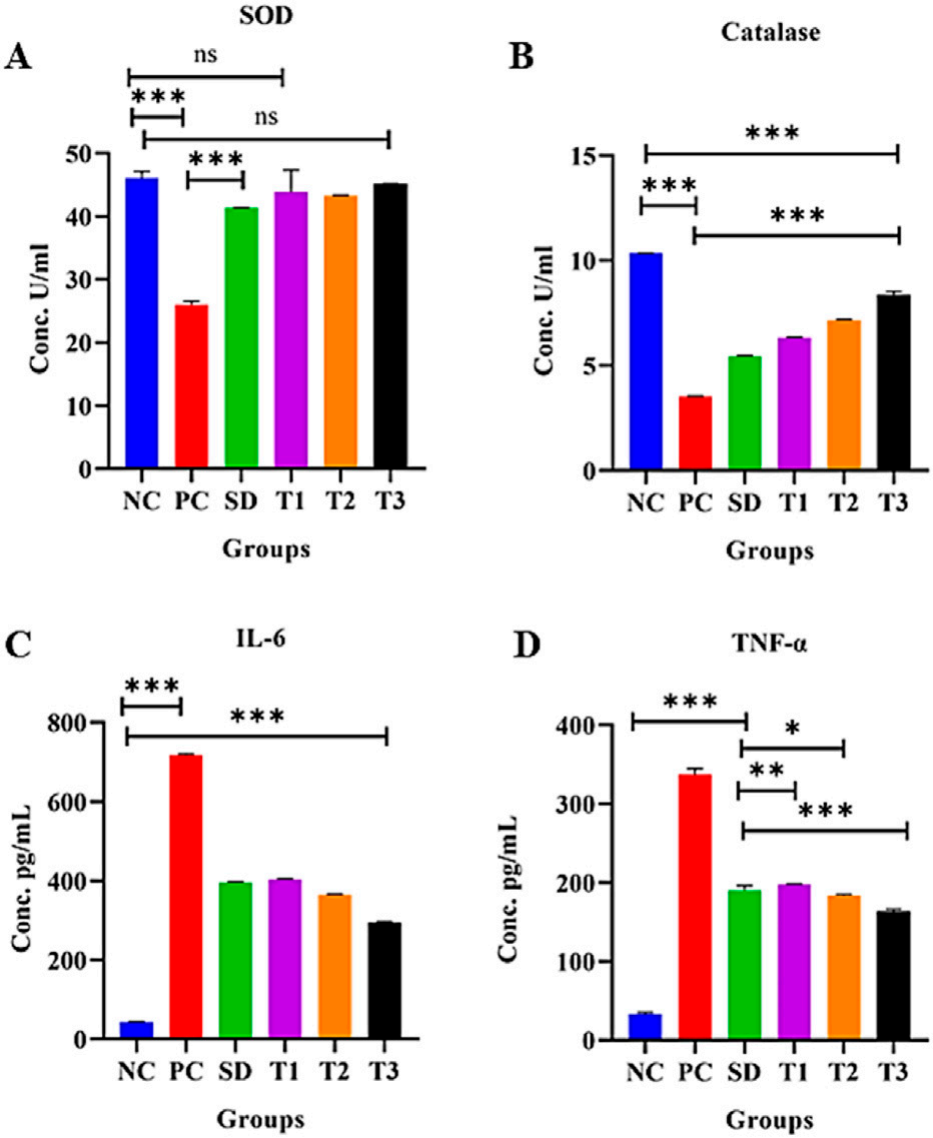
Effect of green synthesized CuO nanoparticles of the *B. amplexicaulis* root extract on **(A)** SOD; **(B)** catalase; **(C)** IL-6; and **(D)** TNF-α levels. Negative control (NC), positive control (PC), standard dose (SD), plant extract (T1), low-dose nanoparticles (T2), and high-dose nanoparticles (T3). Significance value: *p <0.05, **p < 0.001, and ***p < 0.0001. Comparison of the disease group with other groups.

A significant decrease in superoxide dismutase (SOD) (U/mL protein) levels (*p* < 0.001) was observed in the positive control group compared to the negative control group. Conversely, a significant increase in SOD levels (*p* < 0.001) was noted in the other treatment groups. However, there was a nonsignificant difference (*p* < 0.001) between the negative control and treatment 1 group, as well as between the negative control and treatment 3 group, as shown in Figure 14A.

##### 3.8.5.17 Effect of green synthesized CuO nanoparticles from the *B. amplexicaulis* root extract effect on catalase activity in liver tissue

The statistical analysis revealed a significant decrease in catalase (CAT) (U/mL protein) levels (*p* < 0.001) in the positive control group compared to the negative control group. In contrast, the treatment groups demonstrated a significant increase in catalase levels (*p* < 0.001), as shown in Figure 14B.

##### 3.8.5.18 Effect of the treatment of green-synthesized CuO nanoparticles from the *B. amplexicaulis* root extract on IL-6 levels

The statistical analysis of IL-6 levels showed a significant increase (*p* < 0.001) in the positive control group compared to the negative control group. In contrast, a significant decrease in IL-6 levels (*p* < 0.001) was observed in the treatment groups compared to the positive control group. Specifically, a notable reduction (*p* < 0.001) in IL-6 levels was observed across three treatment groups, as shown in Figure 14C.

##### 3.8.5.19 Effect of the treatment of green synthesized CuO nanoparticles from the *B. amplexicaulis* root extract on TNF-α levels

The statistical analysis revealed a significant increase in TNF-α levels in the positive control group compared to the negative control (*p* < 0.001). Conversely, a significant decrease in TNF-α levels (*p* < 0.001) was observed in the treatment groups compared to the positive control group, as shown in Figure 14D.

##### 3.8.5.20 Effect of green synthesized CuO nanoparticles from the *B. amplexicaulis* root extract effect on histopathological examination

###### 3.8.5.20.1 Liver

**FIGURE 15:**
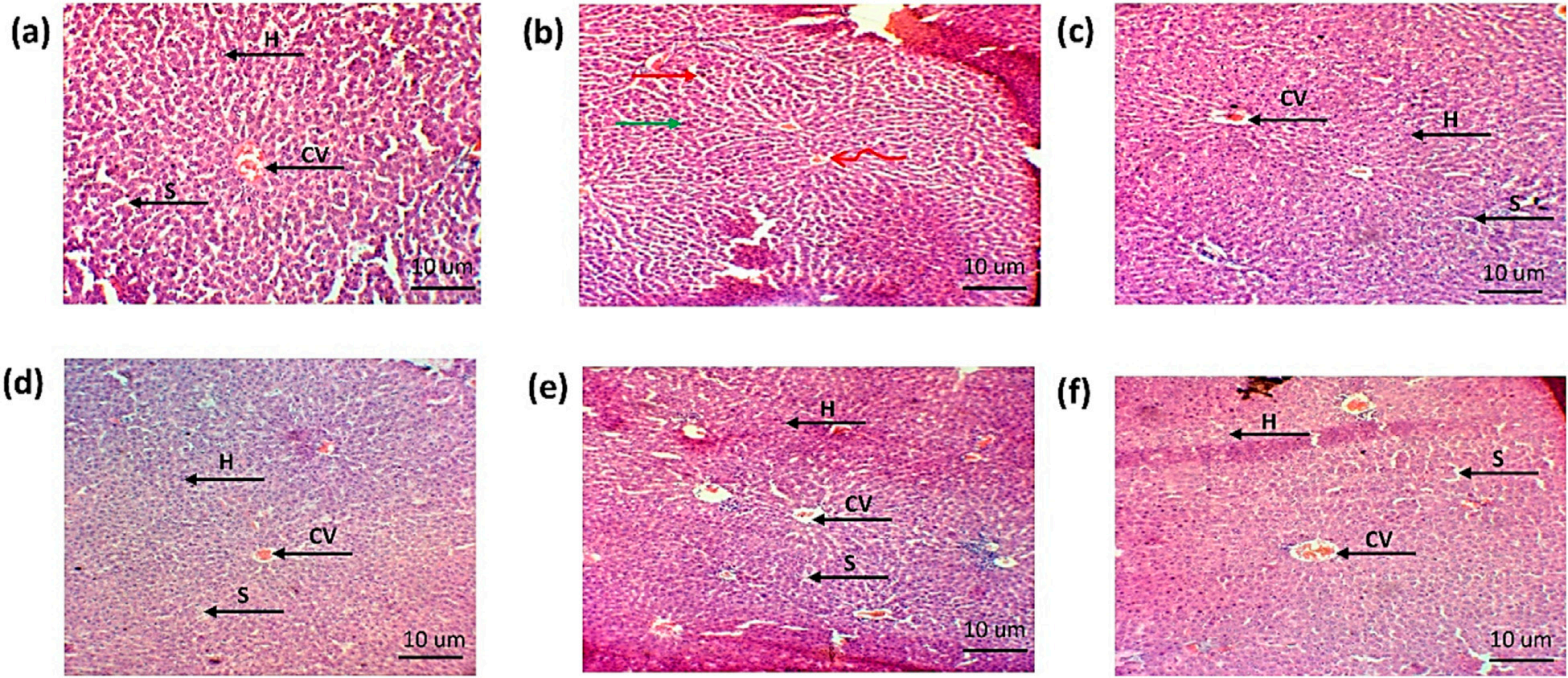
Histopathological analysis of liver **(A)**. Negative control group **(B)**. Positive control **(C)**. Standard **(D)**. Simple extract **(E)**. Low-dose NPs **(F)**. High-dose NPs. Green arrow: hepatocytes vacuolated; curved arrow: sinusoids degenerated; arrow H: hepatocyte. CV: central vein. S: sinusoids.

The histopathological examination of the liver showed hepatic cell necrosis, infiltration of monocytes, and vacuolization in the arthritic control group. However, the treated groups showed a reduction in the monocyte infiltration and vacuolization, as shown in Figures 15A–F.

###### 3.8.5.20.2 Kidney

**FIGURE 16:**
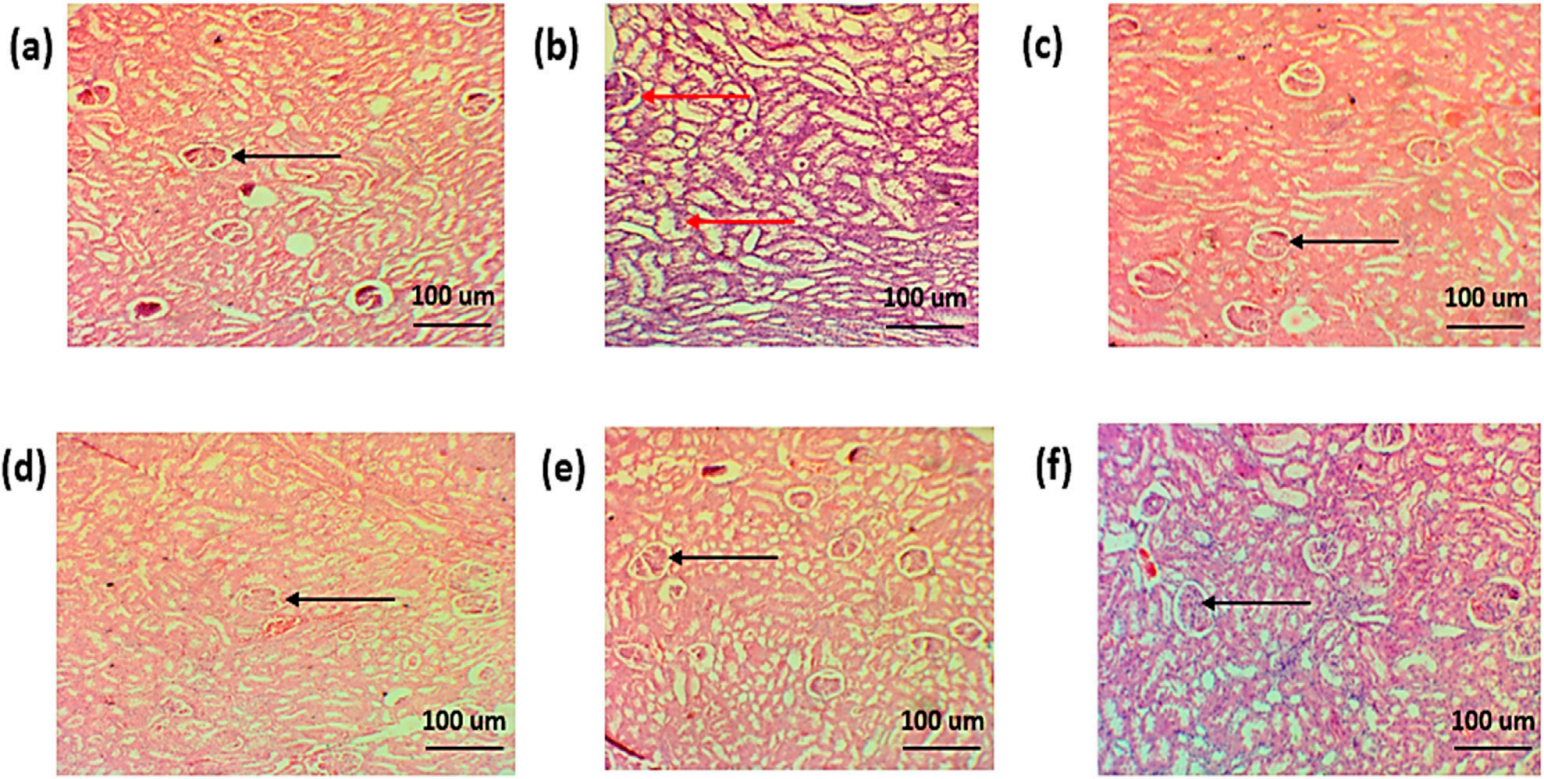
Histopathological analysis of the kidney **(A)**. Negative control group **(B)**. Positive control **(C)**. Standard **(D)**. Simple extract **(E)**. Low-dose NPs **(F)**. High-dose NPs. Red arrow: dilation of the glomerulus. Black arrow: normal morphology of glomeruli.

The histopathological examination of the kidney of normal group showed normal renal parenchyma. The histopathology of the standard treatment group showed damaged renal parenchyma, necrosis, and congestion. Infiltration of inflammatory cells was also observed. However, in all other groups, less damage to renal parenchyma, less congestion, and less infiltration of inflammatory cells were observed, as shown in Figures 16A–F.

###### 3.8.5.20.3 Ankle joint

**FIGURE 17:**
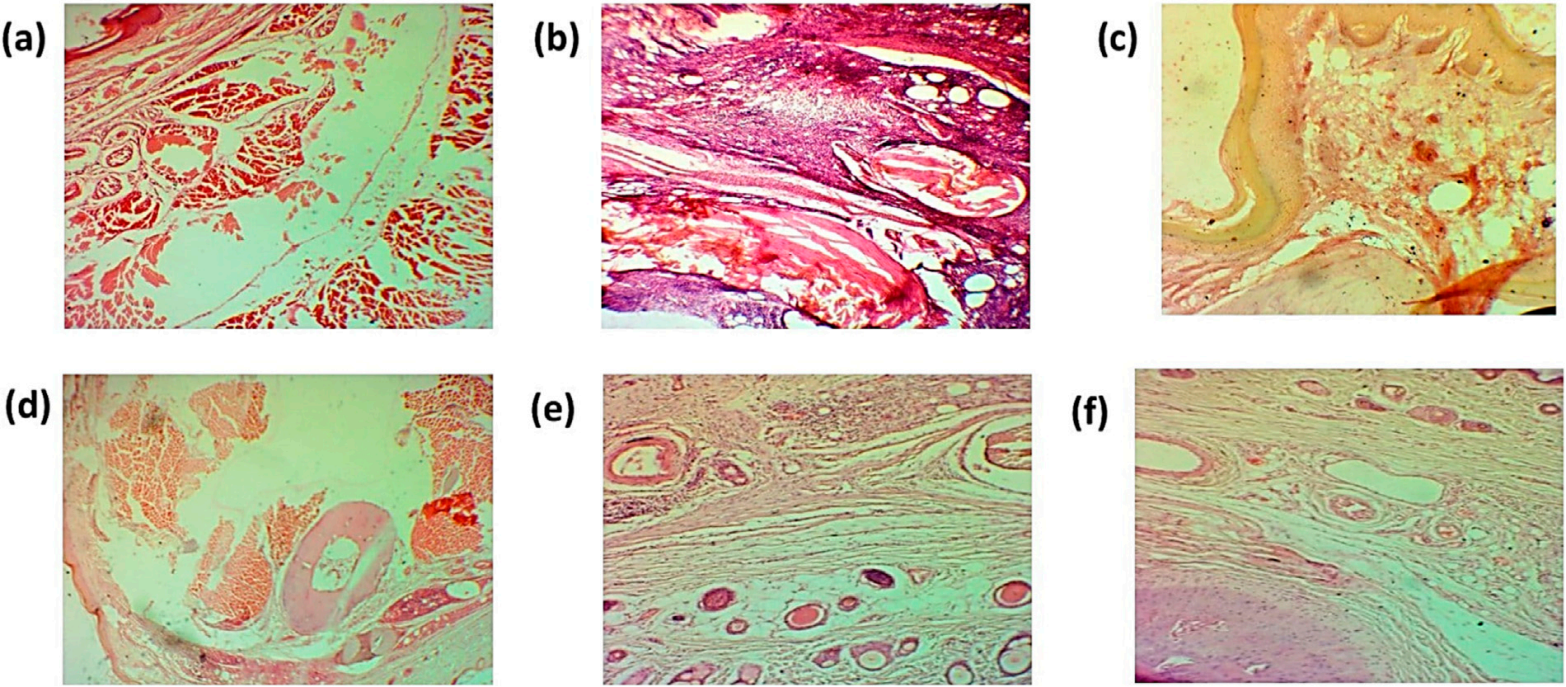
Histopathological analysis of the ankle joint **(A)**. Negative control group **(B)**. Positive control **(C)**. Standard **(D)**. Simple extract **(E)**. Low-dose NPs **(F)**. High-dose NPs.

The histopathological examination of the ankle joint shows mononuclear cell infiltration, damage of bone, and hyperplasia of the synovium. However, histopathology of treated groups showed a decrease in mononuclear cell infiltration, as shown in Figures 17A–F.

##### 3.8.5.21 Effect of the green synthesized CuO nanoparticles from the *B. amplexicaulis* root extract on the radiological examination of paw thickness

**FIGURE 18:**
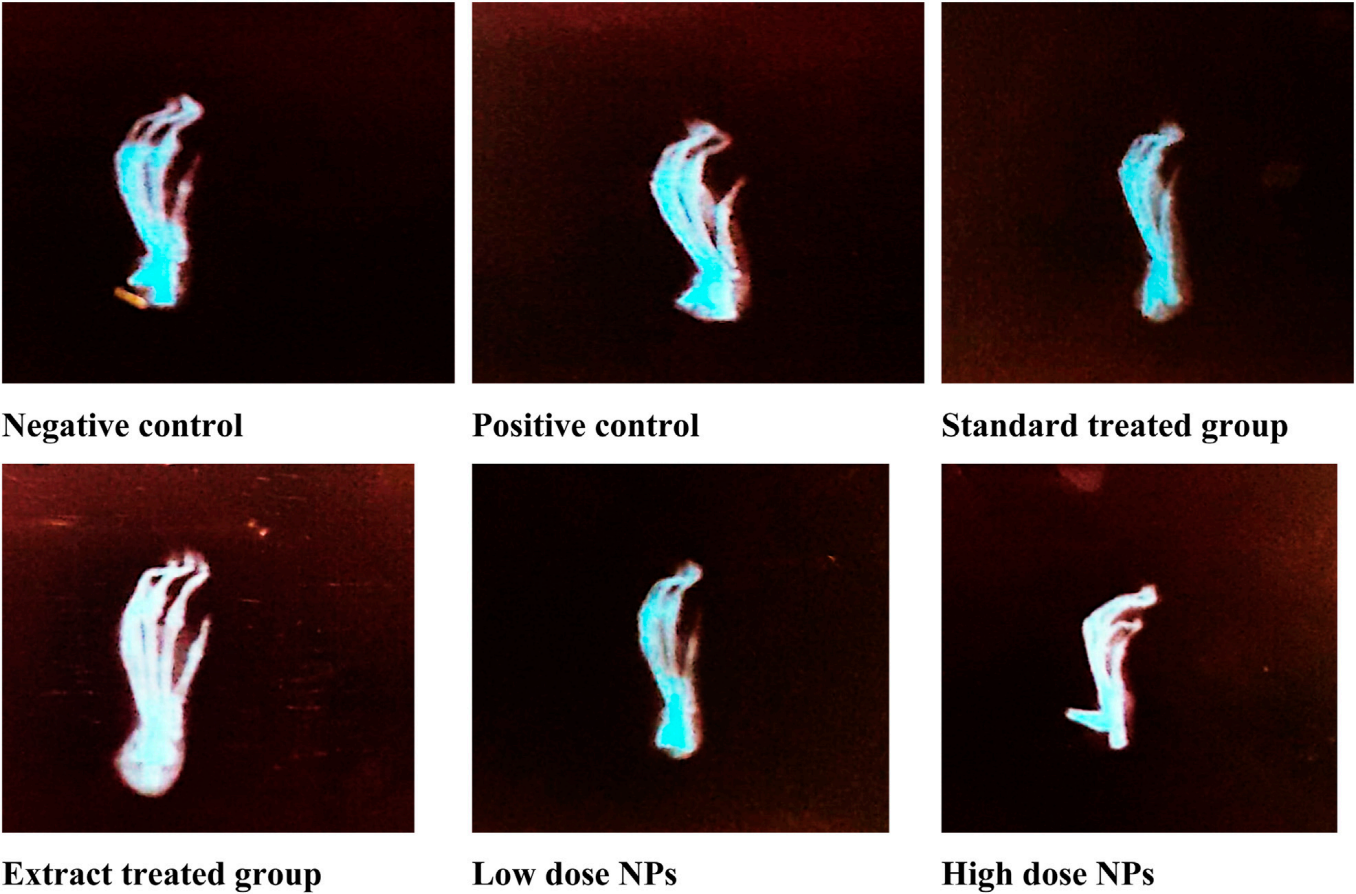
Radiological examination of experimental rats.

Radiological X-ray examination depicted marked inflammation in the arthritic group (B) compared to healthy rats. Radiological examination of arthritic rats showed swelling of soft tissues, narrowing of joint space, ankylosed joints, periosteal reaction at the metatarsal area, and osteolysis inconsistency with paw swelling and arthritic score compared to the healthy group. The treatment with both the plant extract and nanoparticles showed attenuated effects; but nanoparticles showed better results than the plant extract dose. Moreover, high-dose nanoparticles exhibited better effects on inflammation than low-dose nanoparticles. Nanoparticle-treated groups showed a marked reduction in ankylosed joints and less swelling and periosteal reaction at the metatarsal area. The hydroxychloroquine-treated group showed a marked reduction in joint space narrowing and bone damage, along with less swelling of tissues (Figure 18).

## 4 Discussion

Formerly known as *Polygonum amplexicaule, B. amplexicaulis* has been used in folklore medicines against various diseases, with a long history of usage for treating pain, fractures, cardiovascular and cerebrovascular illnesses, and other conditions, and is renowned for its antioxidant, antibacterial, anticancer, antifungal, and cardioprotective properties (Xiang et al., 2011). Therefore, in this study, phytochemical *B. amplexicaulis* extract was phytochemically characterized and evaluated for antioxidant potential *in vitro* and anti-arthritic activity in FCA-induced arthritis in albino Wistar rats.

The estimated carbohydrate content of *B. amplexicaulis* was high. Crude fiber content was estimated to be 8%, and crude fiber could lower the risks of digestive disorders by increasing bowel movements and aid in the increased absorption of trace minerals in the gut (Abolaji et al., 2007; Olowokudejo et al., 2008). Ash content provides an estimation of the mineral content in the plant. The total nutritive value was estimated to be 343.08 kJ/mol. The daily recommended energy value is 1,900–2,900 kcal/day to maintain a healthy weight (Rehman and Adnan, 2018).

The results depicted that *B. amplexicaulis* has a high content of calcium, cobalt, copper, iron, and zinc and a low amount of cadmium and manganese. All these minerals are necessary for bones, blood coagulation, structural stiffness, and as an enzyme co-factor (Rodwell et al., 2015). According to Pathak and Kapil (2004) immunity, cell reproduction, and protein synthesis are all dependent on zinc, oxidation of biomolecules to manage obesity, and for the synthesis of hemoglobin (Thomas and Krishnakumari, 2015; Indrayan et al., 2005). Cobalt aids in the synthesis of cobalamin (vitamin B12) and vitamin absorption (Yamada, 2013). These minerals strengthen the immune system and act as antioxidants by lowering lipid peroxidation.

Preliminary phytochemical screening of *B. amplexicaulis* showed the presence of alkaloids, amino acids, cardiac glycosides, betacyanins, carbohydrates, flavonoids, fixed oils, phenols, phlobatannins, quinones, tannins, and terpenoids. Flavonoids and phenols are considered valuable due to their attributed antioxidant potential. The free radical scavenging nature of flavonoids and phenolics makes them valuable in oxidative stress and has efficacy in cardiac and inflammatory disorders (Doughari, 2012).

HPLC techniques were used for the quantification of flavonoids and phenolic compounds. Quercetin, vanillic acid, caffeic acid, chlorogenic acid, gallic acid, and coumaric acid were detected in *B. amplexicaulis*, owing to its antioxidant, hepatoprotective, anti-pyretic, analgesic, neuroprotective, and anti-inflammatory properties due to its free radical scavenging capabilities (Ying et al., 2013; Naveed et al., 2018; Espíndola et al., 2019; Pei et al., 2016).

The FTIR spectrum of the *B. amplexicaulis* extract indicated the presence of different functional groups at different frequency ranges. The spectrum showed that the plant is rich in antioxidants. The carboxyl group at 3,500–2,400 cm^−1^ indicated the presence of flavonoids in the plant. The presence of an O-H group at 1,390–1,310 cm^−1^ indicated the presence of phenols, owing to the antioxidant potential of the plant.

*In vitro* DPPH bioassay was performed to validate and scrutinize the antioxidant capacity of the *B. amplexicaulis* extract, which showed the significant free radical DPPH scavenging ability of the plant (Szabo et al., 2007).

CuO nanoparticles were synthesized using the green synthesis method, which is cheap, less harmful to the environment, and also because copper oxide has various pharmacological activities including antioxidant, anti-tumor, and antibacterial effects (Maqbool et al., 2017). These nanoparticles were characterized with SEM and particle size and zeta potential techniques, which confirmed that NPs were predominantly spherical and non-crystalline with a zeta size of 186.8 nm and strong anionic zeta potential of −9.23 mV, indicating good stability. The FTIR analysis of CuO NPs showed the presence of various functional groups including O-H, C-O, N-O, and C-N, which indicated the presence of phenolic, ketonic, nitro, and amino groups, owing to the antioxidant potential of nanoparticles (Clogston and Patri, 2011; Al-Kalifawi, 2016).

In the present study, the hydroalcoholic extract and CuO nanoparticles of *B. amplexicaulis* were evaluated for its anti-arthritic activity in FCA-induced arthritis albino Wistar rats. Post-arthritic oral treatment with hydroxychloroquine, extracts, and nanoparticles significantly reduced the paw inflammation and arthritic score in arthritic rats, with the higher dose of nanoparticles producing remarkable effects compared to hydroxychloroquine and plant extract, which suggest the possible anti-arthritic effect of CuO nanoparticles. These nanoparticles also significantly normalized hematological parameters such as red blood cells, white blood cells, Hb, and platelets, indicating an ameliorative effect on arthritis conditions. Anemia is an indication of rheumatoid arthritis. Various factors, including the suppression of bone marrow, ineffective erythropoiesis, and GIT bleeding, may cause anemia (Bowman, 2002). Studies reported that arthritic patients commonly experienced anemic conditions and observed a link between the severity of arthritis symptoms and the degree of anemia. Treated RA patients, having improved anemia, also showed improvement in the quality of life (Wilson et al., 2004). The WBCs are an essential immune system marker, which is associated with inflammation induction and other infectious diseases (Bowman, 2002). Induction of pro-inflammatory cytokines into the injured area and stimulation of the immune system can be indicated by an increased platelet count and WBC level in RA patients (Sattar et al., 2003).

The immune organs such as the thymus and spleen of arthritic rats were also observed at the end of the study and weighed. CuO nanoparticles showed better results than the standard drug and extract of *B. amplexicaulis* roots. CFA-induced arthritis significantly upregulated the serum levels of cytokines, including TNF-α and IL-6, accompanied by an elevation in circulatory inflammatory cells, which can be confirmed through the increased weight of the thymus and spleen (Banji et al., 2014; Akramas et al., 2015).

In the present study, CuO nanoparticles reduced the levels of RA and CRP in arthritic rats. The CRP is considered one of the essential systemic inflammatory markers. Several studies reported a conflicting relation of RA diagnosis with the CRP level. An increased CRP level was found in patients before RA diagnosis (Masi et al., 2001), and one study showed an increased level of CRP in male and female blood donors within 2 years of RA diagnosis but changes in the CRP level did not correlate primarily with the prediction of RA incidence (Shadick et al., 2006). The level of autoantibodies, such as RF linked to the severity of the disease, is partly based on structural damage. The detection of the RF in the serum is assumed as an independent predictor of the structural deterioration of joints (Laurent et al., 2011).

The biochemical analysis of liver function markers of arthritic rats demonstrated an increase in ALT and AST levels, indicating free radical-mediated hepatic injury (Ijaz et al., 2022). The elevated level of these cellular enzymes can be indicative of bone and organ impairment. The generation of mediators was affected by increased ALT and AST levels and, hence, accelerates the bone and organ damaging effects in RA patients (Rehman and Lane, 2001; Coelho et al., 2004). The results of the present study showed the possible anti-arthritic ability of the *B. amplexicaulis* extract and CuO nanoparticles, along with protecting bone loss and organ damage by reducing the levels of these indexes, which not only indicated the antioxidant effect but also hepatoprotective activity of these nanoparticles, which was good compared to the standard treatment drug. Hydroxychloroquine showed an elevation in the levels of these indexes, indicating that this drug is not hepatoprotective and has liver-associated side effects. However, nonsignificant alterations of renal function markers such as creatinine and urea were observed in the present study contrary to previous studies (Mishra et al., 2011).

The persistent dysregulated generation of IL-6 plays a vital function in RA development and other autoimmune disorders. The results of the present study showed an upregulation in IL-6 expression in arthritic rats, while hydroxychloroquine extract and CuO nanoparticle-treated arthritic animals exhibited a decreased level of IL-6 expression.

The results of the present study demonstrated the marked effect of treatments on TNF-α expression in arthritic rats. Hydroxychloroquine, extract, and CuO NPs showed the suppression of TNF-α compared to arthritic control. CuO NPs showed better suppression of TNF-α levels than other treatment groups. TNF-α is also believed to cause connective tissue damage by inducing PGE2 and collagenase synthesis (Vasanthi et al., 2007).

Histopathological examination was performed to check the effect of biosynthesized CuO NPs on the ankle joint, liver, and kidney. The arthritic group showed an increase in mononuclear cells, formation of pannus, bone destruction, and hyperplasia of the synovium in ankle joint (Figure 17). The results revealed a decrease in mononuclear cells and reduction in bone destruction. Liver histopathology also showed a significant number of inflammatory cells, hepatic cell congestion, and necrosis, which were significantly reduced by treating arthritic rats with green synthesized CuO NPs (Figure 15). The kidney displayed the infiltration of inflammatory cells and necrosis in the standard group, which decreased to a lower degree of infiltration and necrosis in other treated groups (Figure 16).

In this study, the findings of radiographic changes of arthritic rats showed swelling of soft tissues along with joint space narrowing, which depicts bone damage in arthritic experimental animals (Ekambaram et al., 2010). The use of hydroxychloroquine in adjuvant-induced arthritic rats showed protection against the damaging effects of arthritis. Arthritic groups treated with the *B. amplexicaulis* extract and CuO NPs at a high dose revealed significant prevention against bone damage and soft tissue swelling observed at the end of the study. CuO NP-treated groups showed better results than plant extract-treated groups. Gross macroscopic observations were in concordance with radiographic findings, as reported in previous studies (Ananthi et al., 2017).

## 5 Conclusion

From the findings, we can conclude the probability of using green-synthesized copper oxide nanoparticles as an alternative treatment for RA, instead of using different therapeutic approaches, due to its antioxidant and anti-inflammatory potential. The given data showed that green-synthesized copper oxide nanoparticles not only significantly affect the rheumatoid arthritis parameters and hematological parameters but also decrease the kidney and liver markers. Inflammatory markers that are directly linked to antioxidants also decrease with the treatment of green synthesized CuO NPs. The histological examination revealed a marked decrease in the number of inflammatory cells in the ankle, kidney, and liver and especially showed significant reduction in pannus formation and bone destruction in green synthesized NP-treated groups. Thus, it was observed that green synthesized copper oxide nanoparticles from *B. amplexicaulis* roots showed better results.

## Data Availability

The raw data supporting the conclusion of this article will be made available by the authors, without undue reservation.
